# Understanding the prevalence of bear part consumption in Cambodia: A comparison of specialised questioning techniques

**DOI:** 10.1371/journal.pone.0211544

**Published:** 2019-02-20

**Authors:** Elizabeth Oneita Davis, Brian Crudge, Thona Lim, David O’Connor, Vichet Roth, Matt Hunt, Jenny Anne Glikman

**Affiliations:** 1 San Diego Zoo Institute of Conservation Research, Escondido, CA, United States of America; 2 University of Bristol, Department of Archaeology and Anthropology, Bristol, United Kingdom; 3 Free the Bears, Phnom Penh, Cambodia; 4 National Geographic Partners, NW, Washington DC, United States of America; 5 The Faculty of Biological Sciences, Goethe University, Frankfurt am Main, Germany; University of Delhi Department of Environmental Studies, INDIA

## Abstract

The trade in bear parts for medicine and for status is a conservation challenge throughout Asia. The Asiatic black bear (Ursus thibetanus) and the sun bear (Helarctos malayanus) are endemic to this region, and populations are estimated to have declined throughout their ranges due to widespread illegal killing of bears and trade in parts, combined with loss of habitat. Previous studies have indicated that legislation alone is insufficient to prevent illegal hunting and trade, indicating instead a need to address demand for bear parts and products. We conducted mixed-method surveys in Cambodia to understand the key motivators for individuals to consume bear parts, and to understand whether specialised questioning techniques are applicable in this context. Bear part use is illegal in Cambodia and may therefore be considered a sensitive behaviour, in that individuals may be reluctant to admit to it. To counteract possible biases, four specialised questioning techniques were used in this study: randomised response technique (RRT), unmatched count technique (UCT), nominative technique (NT), and false consensus bias (FCB). All four methods serve to shield a respondent’s admittance of a sensitive behaviour from the interviewer. The results presented here show that great variability exists in anonymous methods’ efficacy in certain contexts. However, the results overall indicate that individuals in Cambodia are under-reporting their consumption of bear parts when directly asked, and that the prevalence of bear part use in Cambodia may be as high as 15% of the population, representing a significant conservation challenge.

## Introduction

The world is currently in the midst of a biodiversity crisis [1, 2]. Use of animal products in traditional medicine is a major driver of the biodiversity crisis throughout the world [3, 4, 5, 6]; however, wildlife products for medicinal purposes have a long history of use, and in many cases is an integral component of human cultures [7, 8]. Use of animals for medical purposes is well-known to be deeply ingrained throughout Asia [8]. In China some have argued that the use of traditional medicine may be declining as Western medicine grows increasingly accessible [9], and this may be true within Southeast Asia as well. Nonetheless, use continues to be ubiquitous throughout the region [8].

Bear parts have been used throughout Asia for thousands of years [10, 11]. Bear paws were recorded as being found on imperial banquet tables as early as the Han Dynasty (206 BC to 220 AD) [12], and they have maintained their role as a high-status dish in Chinese society [13, 14]. Bear gallbladder and bear bile are components of traditional medicine (TM), and are used to treat a variety of ailments [15], including fever, toothache, and stomach ailments [16]. At present it is unknown whether bears are a traditional source of medicine in Cambodia, whether their use is a recent trend brought in from neighbouring Vietnam and China, or whether use of bear parts in Cambodia is some combination of the two.

What is known is that present levels of trade in bear parts in Asia are not sustainable and have been negatively impacting bear populations. This is especially true for sun bears (*Helarctos malayanus*) and Asiatic black bears (*Ursus thibetanus*). Current estimates are that these bear populations have declined by approximately 30% over the past 30 years [17, 18]. Although Appendix I of CITES prohibits international commercial trade of bear parts, and most Asian countries have prohibited trade of bear parts within their borders, trade continues to be widespread (e.g. Cambodia; [19], Lao PDR; [20], and Vietnam; [21]). As regulation alone has had little documented effect on this trade [22], additional means are being employed to halt the trade, and reduce or change demand. For example, drivers of bear part use among certain groups are beginning to be explored using social science techniques, with the intent of using that information to inform behaviour change campaigns [23, 24].

However, direct questions about sensitive behaviours, such as bear part use, can sometimes result in deceit from participants due to worries about getting punished for an illegal action, and/or social stigma that might be associated with the behaviour [25]. Killing bears is illegal in most Asian countries, and use of bear parts is, by extension, often illegal [26], as many users of bear parts will either by necessity or choice source their bear products illegally [23, 24]. Therefore, alternatives to direct questioning, such as randomized response technique (RRT), unmatched count technique (UCT) and nominative technique (NT), may provide a better picture of use by enabling respondents to answer confidentially and provide more truthful responses [25]. RRT uses a randomisation tool, most commonly a die, to integrate probability into the given response of the respondent. This probability ensures that the “true” response of the interviewee is never known by the interviewer, thus ensuring complete anonymity. In UCT, responses are shielded by asking respondents to state the number of actions performed, some of which may be considered sensitive. In NT, respondents are asked to discuss the behaviour of their social group, rather than their own behaviour, thus allowing the respondent to keep their own behaviour hidden. Additionally, false consensus bias (FCB) can assist in estimating the prevalence of certain behaviours in a population by using respondents’ own perceptions of a behaviour’s prevalence among their social group as an indicator of the respondent's own behaviour [27]. In a conservation setting, this technique has been paired with RRT as an additional measure of behaviour prevalence [28], under the assumption that individuals who engage in a sensitive behaviour will estimate a greater proportion of the population as being involved in that behaviour [29].

All four methods are beginning to be applied to conservation settings, though their use is rare in Asia. In the published literature, RRT, paired with an analysis of potential FCB, has been proven to be successful in Taiwan, when asking respondents about illegal carnivore killing [29]. Prevalence estimates derived from RRT responses were found to be much higher than from direct questions for the sensitive behaviour, and correlated with an observable false consensus effect. UCT has not been used widely in Asia, but in Tanzania UCT appeared successful in encouraging respondents to answer truthfully about poaching [30]. One previous study of UCT has been conducted in Cambodia, though the method was found to be unsuccessful, supposedly because the behaviour in question had a low “true” prevalence within the population [31]. Additionally, an online survey conducted by Hinsley et al. [32], which included individuals from Indonesia, Malaysia, and Japan, found no evidence of an effect when using UCT in comparison to direct questions. There has been no published conservation research using NT in Southeast Asia. More generally, NT isn’t prevalent in conservation studies, apart from St. John et al. [25], who found it to be ineffective in their study about illegal fishing in Wales. They hypothesised this inefficacy was due to the respondents in their study being unfamiliar with their friends' illegal fishing behaviour, rather than any issues with implementation.

This study tests whether these methods can be successfully employed in Cambodia for asking sensitive questions about illegal use of wildlife parts, especially as there are no published instances of any of these specific SQT methods being used in Cambodia to date. As the mechanics affecting social desirability vary widely between demographic and cultural groups [33], it is expected that SQTs will have variable success in varying demographic and cultural contexts. Therefore, the success/failure of some of these methods in other contexts [25], may not indicate parallel success/failure of these methods in Cambodia. For example, lack of a sufficiently communicative social network could potentially affect NT [25], and thus NT may be more effective in Southeast Asia where more homophilic social networks, e.g. the village, are abundant [34].

In addition to the legality concerns, illegal wildlife part use may also be socially undesirable in certain contexts, thus making it superficially analogous to such behaviours as illegal drug use e.g.[35]. SQTs have been widely used in understanding the prevalence of illegal drug use among populations, and thus they have been argued to be applicable in understanding other sensitive conservation-negative behaviours, such as poaching [30]. While not widely used in the illegal wildlife trade literature, SQTs could provide the most accurate measure of wildlife part use prevalence. However, although these methods may have been successful in Western contexts, quantitative questions are well-known to be context-dependent, and questioning techniques that work in certain cultures and among certain demographics may not work in others [36]. For example, a study performed in Cambodia that utilised unmatched count technique (UCT) to understand the prevalence of illegal bird hunting and egg collection was not successful, perhaps due to the low behaviour prevalence, but contextual challenges cannot be ruled out [31]. To our knowledge, no other published SQT study has been performed in a Southeast Asian context, for any other sensitive issue. Thus, RRT, NT, and FCB are currently untested in Southeast Asia, including Cambodia.

The main objective of this study is to provides the first quantitative measure of the extent of bear part use in Cambodia, as well as the levels of deceit involved when individuals in Cambodia discuss this illegal behaviour. A secondary important aim is to address the gap in the use of SQT, by assessing the efficacy of the four methods in a Southeast Asian country, as well as testing them for soliciting truthful answers in the use of illegal wildlife parts. The scope of this study is such that elucidating the historic and current role of bears in traditional Cambodian medicine is impossible; rather the study’s objective is on gathering information that can be directly beneficial to conservationists working within Cambodia. Comparing the estimates obtained through these specialised techniques to the direct responses provides an indication of the true prevalence of bear part consumption throughout Cambodia. This baseline measurement can inform where future demand reduction campaigns within Cambodia should be implemented, as well as the potential, projected impacts of behaviour change.

## Materials and methods

### Study site

**Fig 1:**
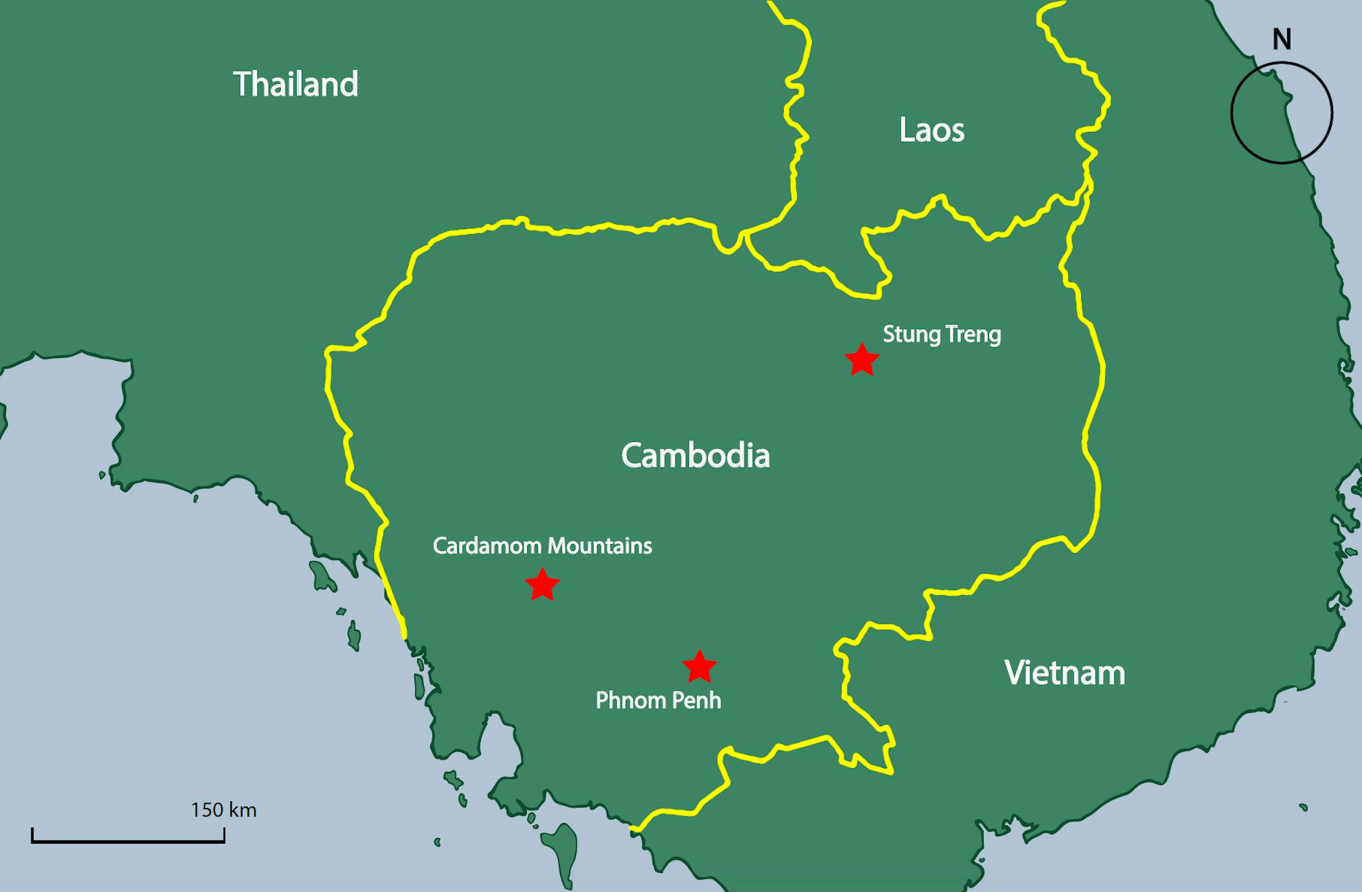
A map of Cambodia. With Stung Treng, Phnom Penh, and the Cardamom Mountains marked. (Map drawn by J. Knox.).

Surveys were conducted in three socio-economically and geographically distinct areas: Phnom Penh City, Stung Treng Province, and the Cardamom Mountains area (Fig 1). Phnom Penh is the capital city of Cambodia and is a densely populated urban area with an estimated population of 1.5 million. Stung Treng, a province in northeast Cambodia, was chosen because it is rural, and relatively ethnically diverse, with a substantial population of Lao and other ethnic minority groups. Nonetheless, as is common throughout Cambodia [37], most of the population in all three locations identified as Khmer (S1 Data).

Surveys were also conducted in communities surrounding the Central Cardamom Mountains National Park, part of a large protected landscape in southwest Cambodia, which harbours a potentially regionally significant population of sun bears [38]. Currently, there is evidence to suggest that Stung Treng and the Cardamom Mountains may be “source” sites of illegal animals and animal parts [39, 40].

### Survey instrument

This study used a questionnaire containing only closed-questions (see S1 Data), which was presented in an interview format, to ensure that questions were understood and answered as intended. The survey instrument was prepared in English with input from local and international experts. The questionnaire was translated to Khmer and back-translated to English by a professional translation service. The back translations were crosschecked against the original English to ensure accuracy. The survey instrument was pre-tested in Phnom Penh and final adjustments were made accordingly.

In the survey, unmatched count technique (UCT) was asked first, followed by nominative technique (NT) and false consensus bias (FCB) questions. These specialised questioning techniques (SQTs) were, in theory, less conspicuous. They were not separate questions and did not require separate “training”. The final SQT to be used was the randomised response technique (RRT) which required additional instructions for respondents and was therefore believed to be more conspicuous and time-consuming. The survey was found to take between 20 to 30 minutes, depending on the interviewer’s skill. The five SQT questions used within the survey, as well as the order they were in, are depicted below in Table 1.

**Table 1:** The five questions used, in order of appearance in the survey.

**Unmatched Count Technique (UCT)**	
How many of the following have you done in the past 12 months?	
*A*	*B*
Cut yourself by accident	Cut yourself by accident
Take a painkiller	Take a painkiller
Use herbal medicine	Use herbal medicine
Take bear bile	Go to the hospital in the US
Go to the hospital in the US	
**Nominative Technique (NT)**	
a. How many of your close friends do you know for certain have used/consumed bear parts for medicine or other purposes? *(If more than one friend has used bear parts*, *randomly nominate one of them*.*)* b. Other than you, how many other people do you believe know that the nominated friend has used bear parts or products for medicine or other purposes?	
**False Consensus Bias (FCB)**	
Thinking of your closest family and friends, what percentage (between 0–100%) of them do you think use bear parts or products for medicine or other purposes? *Please circle* *one* *below–if you don’t know*, *please guess*. 0–20% 21–40% 41–60% 61–80% 81–100%	
**Direct**	
Have you ever consumed/used any of the following bear parts or products? Ex: bear paw soup, bear bile, etc (answered *yes* or *no*)	
**Randomised Response Technique (RRT)**	
Have you ever used bear bile or gallbladder?	

To compare with the SQTs, the direct estimate was calculated as being all self-stated users of bear bile and bear gallbladder, as these two terms are used interchangeably for indicating “bear bile use” for medical purposes.

### Sampling method

Stung Treng and Cardamom Mountains sampling sites were chosen through cluster random sampling. Districts were first divided by commune, then each commune was then further divided into randomly selected streets where the survey team would perform interviews. Each research assistant walked a different street each day, and surveyed every 4^th^ household on their left. This sampling was also used due to the scattered distribution of households in this rural area. One person in each household was surveyed, and all respondents were over 18. Male/female respondents were alternated. A total of 1,934 interviews were performed in all three sites (Stung Treng: n = 636; Cardamom Mountains: n = 649; Phnom Penh: n = 649) (**Table 2**).

**Table 2:** Demographics.

*Demographics*		*Stung Treng*			*Cardamom Mountains*			*Phnom Penh*		
**Gender**	**Male**	48.8%	(n = 312)		48%	(n = 311)		50%	(n = 325)	
	**Female**	51.2%	(n = 324)		52%	(n = 338)		50%	(n = 324)	
**Age**	**Range**	18–75			18–80			18–86		
	**Average**	37			37			31		
	**Median**	35			34			26		
**Education**	**Common Highest Level**	Secondary school	27.3%	(n = 175)	Primary school	44.1%	(n = 286)	University (Bachelor’s)	33%	(n = 212)
Religion		Buddhist	82.5%	(n = 529)	Buddhist	75.5%	(n = 529)	Buddhist	92%	(n = 598)

In Phnom Penh, five public parks were chosen as sampling sites by the Phnom Penh Capital Hall because household sampling was deemed to be biased towards lower income individuals. Public parks in Phnom Penh are used by a cross section of society. However, they are sparsely populated during the day, thus sampling effort was limited to the evenings and weekends. As in the two other areas, males and females were alternated between, excluding any person who had been living in Phnom Penh for less than two years. This was to adequately reflect “true” residents of Phnom Penh, rather than transient individuals [41]. Although it is possible that interviewed individuals in the Cardamom Mountains or Stung Treng were recent migrants, it is less likely, as those interviews were conducted at established households, rather than public areas.

Potential interviewees were informed that the survey was completely confidential and anonymous, and that they could refuse to answer any question or stop the interview at any time. Verbal consent was considered given if interviewees indicated understanding and acceptance of the terms, according to a set verbal consent form that was approved by both ethical boards (IRB and the University of Bristol). Written consent was not obtained due to time concerns, additionally some individuals were illiterate. The interview was not allowed to move forward if verbal consent was not obtained. Research assistants were thoroughly trained in understanding this, and the research team leader was always present at field sites to ensure that interviews were conducted to the highest ethical standard. Ethical approval was provided by Miami University Ohio IRB for Human Subject Research (Protocol Number FWA00023676) and the University of Bristol Ethics Board.

### Specialised questioning techniques

In UCT the interviews are split into two groups: treatment or control. Each respondent is asked the question “How many of these activities have you done?”, and shown cards with a variety of activities on them. The control group is shown cards with four non-sensitive activities, while the treatment group is shown cards with the same four non-sensitive activities, as well as an additional sensitive activity, for example poaching. The interviewee will answer with the number of activities they have performed. The difference between the two groups provides an estimate of the prevalence of the sensitive behaviour.

For RRT, the interviewer used a die as the randomisation tool. The interviewee shook the die in a cup (to hide the outcome from the interviewer), and looked at the result. One side of the die was painted red, one green, and the four other sides left blank. If the die came up red, the interviewee was always to answer “no” to the question posed by the interviewer. If the die came up green, the interviewee would always answer “yes”. If the die was blank, the interviewee would answer truthfully. This method ensured that the interviewer was never certain as to whether a response was forced (i.e., the result of a red or green throw), or was truthful [42]. However, RRT has been criticised for having very high variance, and for confusing respondents and sometimes making respondents suspicious [43]. Because of these issues, nominative technique (NT) has been proposed as an alternative [35]. The key difference between RRT and NT is that in NT the sensitive behaviour of interest is not asked about regarding the interviewee, but rather regarding the number of close friends/family of the interviewee who perform the sensitive behaviour. For example, an interviewee may state that they have three close friends who poach. When accounting for duplication, i.e. stated close friends between respondents who are the same individual, the true prevalence of the behaviour in the population can be estimated [25].

As an estimate of behaviour prevalence FCB is simple in execution. Respondents are asked what percentage of their friends they believe to have performed the behaviour in question. This belief is then matched with the number of respondents who have self-stated or been found through SQTs to have performed the behaviour. A comparison of these two estimates allows for ascertaining whether belief in the prevalence of the behaviour is higher among respondents who have used bear parts.

Respondents were also asked to state which questioning technique of RRT and UCT they trusted most, and which they found easier to understand. The parameter of “trust” and “comprehension” were investigated so as to inform the application of these techniques in future SQT studies within Cambodia.

### Statistical analysis

Prevalence estimates from the SQTs were calculated using the following three formulae. Each formula was adapted from published work. The RRT formula used was [21]:

$$\pi =\frac{\left(\lambda -\theta \right)}{s}$$ (1)

In this equation, *π* is the anticipated proportion of the sample who are truthfully admitting to the behaviour. *λ* is the proportion of all answers in the sample that are “yes”, with *θ* standing for the probability of the answer being a “forced” yes (1/6 in our study). Finally, *s* is the proportion of truthful answers (2/3 in our study) [26].

The formula for UCT is calculated by subtracting the mean number given for the control group from the mean number given for the treatment group. This can be represented formulaically as follows [31]:

$$\pi ={\bar{x}}_{A}-{\bar{x}}_{B}$$ (2)

Where *π* is the prevalence estimate, ${\bar{x}}_{A}$ is the average number given by the treatment group, and ${\bar{x}}_{B}$ is the average number given by the control group.

NT uses the following formula to assess prevalence and account for duplication of response [19]:

$$T_{X}=\sum_{j=i}^{n}\frac{A_{j}}{1+B_{j}}$$ (3)

With *T*_*X*_ representative of the estimated number of individuals in a sample size *n* performing the sensitive behaviour. *A*_*j*_ represents the number of rule breakers that the respondent *j* knows, with *B*_*j*_ representing the number of friends, other than *j*, that know of the “nominated friend’s rule-breaking” [35].

FCB was calculated by tallying the number of individuals who said “yes” to each category presented. The category that had the greatest number of responses was deemed to be the estimate of prevalence, as determined by the respondents. Additionally, respondents were separated into users and non-users of bear parts, and FCB responses between groups were compared using a standard chi-squared test.

All data were analysed using the software program R [44] and all figures were created using the package ggplot2 [45]. 95% confidence intervals were calculated for every estimate.

## Results

### Respondent characteristics

The total number of respondents and their demographics are summarized in Table 2. The sample population appears to mirror the overall demographics of Cambodia. Cambodia’s gender ratio is roughly 50:50 [46]. The average age of Cambodia is lower than that obtained here, but that reflects our sampling protocol, in which only adults above the age of 18 were sampled. Buddhism is the dominant religion in Cambodia, and this was reflected in the three sampling sites.

The demographics of Stung Treng, the Cardamom Mountains, and Phnom Penh (total n = 1,934).

As seen in Table 2, all three sites varied in what was the most common highest level of education. Phnom Penh respondents spent more time in formal education compared to the other two samples. This is to be expected since Phnom Penh is the most urban of the three sites, and the interview locations in the Cardamom Mountains were the most rural, with decreased educational access

No measure of income was included, due to the recognized problems of deceit and discomfort that can occur when asking individuals about their income [47]. In addition, during survey design, local experts indicated that asking about income level could be more sensitive than asking about bear bile. Therefore, education and “urban” and “rural” were used as rough proxies of socio-economic status.

### Prevalence estimates by specific location

**Fig 2:**
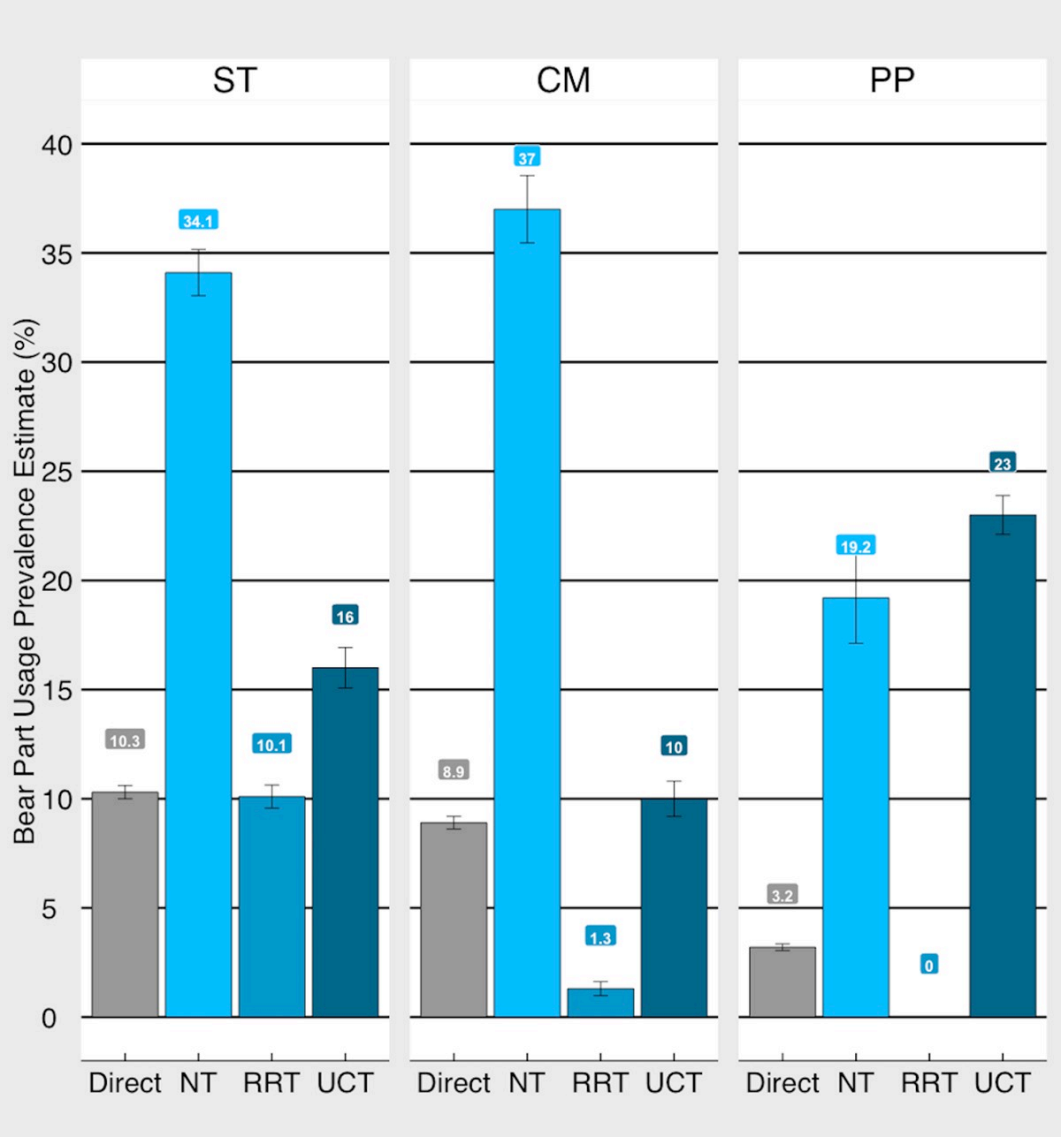
Prevalence estimates. Prevalence estimates of bear part use obtained through specialised questioning techniques (SQTs) and direct questioning for all three field sites (Stung Treng (ST): n = 641; Cardamom Mountains (CM): n = 638; Phnom Penh (PP): n = 649.

The prevalence estimates for bear part use for each specific location are represented in Fig 2. In Stung Treng, direct questioning and RRT showed similar estimates of the prevalence of bear part use, while UCT and NT were both higher. For NT, the prevalence estimate was also higher in the Cardamom Mountains, with every other technique yielding a much lower estimate. In Phnom Penh, the highest estimate was obtained using UCT, but it was not significantly higher than NT.

There was no difference between the results gained from direct questioning and RRT in Stung Treng. In the Cardamom Mountains and Phnom Penh the prevalence estimate obtained using RRT was lower than direct questioning, indicating mistrust and/or misunderstanding of the method.

In addition, the type of parts used between sites was investigated. Differences were found in part use in each site. The less valuable and more perishable parts, such as bear meat, were consumed closer to the source (Cardamoms).

### Prevalence estimates for the country of Cambodia

**Fig 3:**
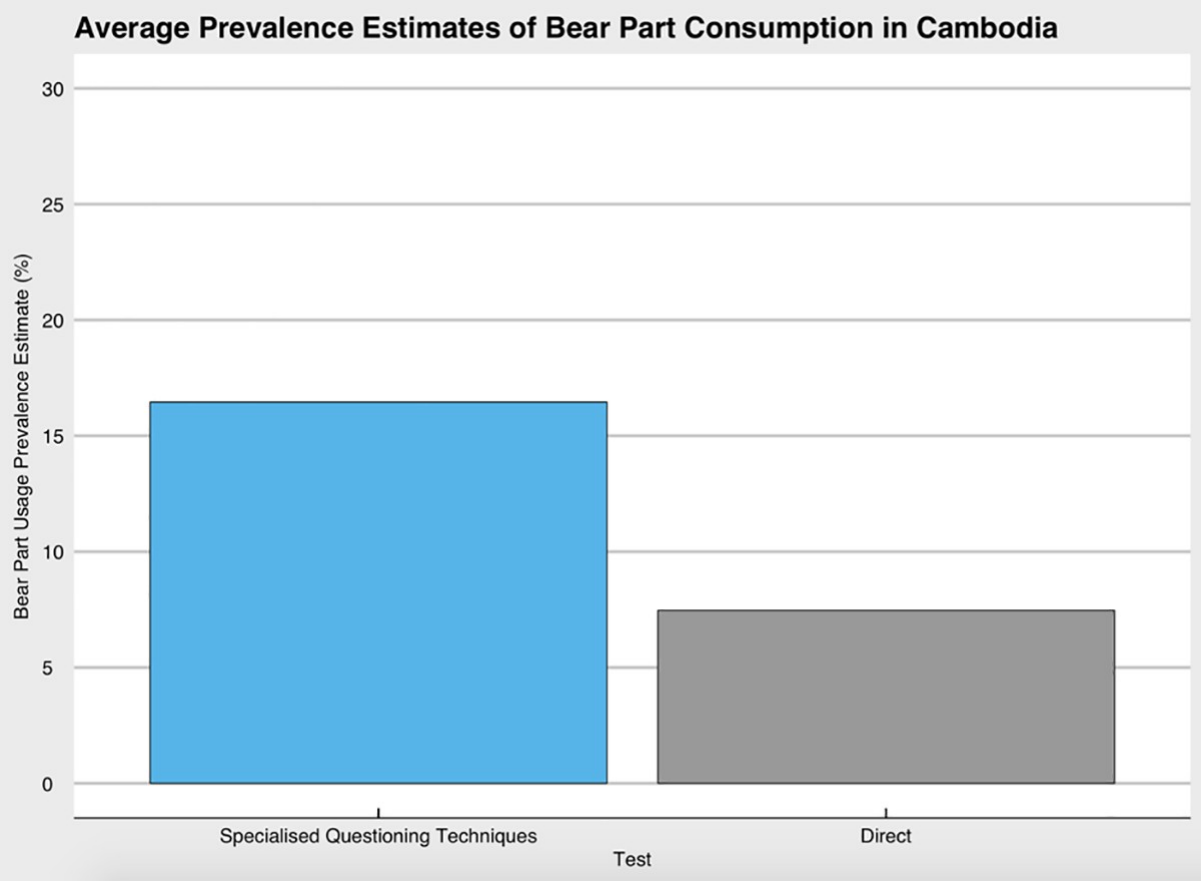
The average of the SQTs and direct questioning prevalence estimates. Calculated from the prevalence estimates obtained at all three sites (n = 1,929).

The estimates of bear part use occurrence and average of the prevalence estimates obtained for the entirety of Cambodia are represented in Fig 3. The prevalence estimates obtained for all three methods for all samples in Cambodia (n = 1,928) were 7.3% for the direct estimate (SE: 0.6%, CI: 6.16–8.48%), 2.3% for the RRT prevalence estimate (SE: 0.89%, CI: 0.02–5.23%), 15.2% for the UCT estimate (SE: 0.35%, CI: 14.5–15.9%), and 27.8% for the NT estimate (SE: 2.9%, CI: 22.1–33.6%). The RRT prevalence estimate obtained is much lower than the other methods, which is consistent with the results found between sites (Fig 3). The SQTs average indicates that over 15% of the population uses or has used bear parts, compared to the direct statements of respondents, which gives a prevalence estimate of roughly 7.5%.

#### False consensus bias

**Fig 4:**
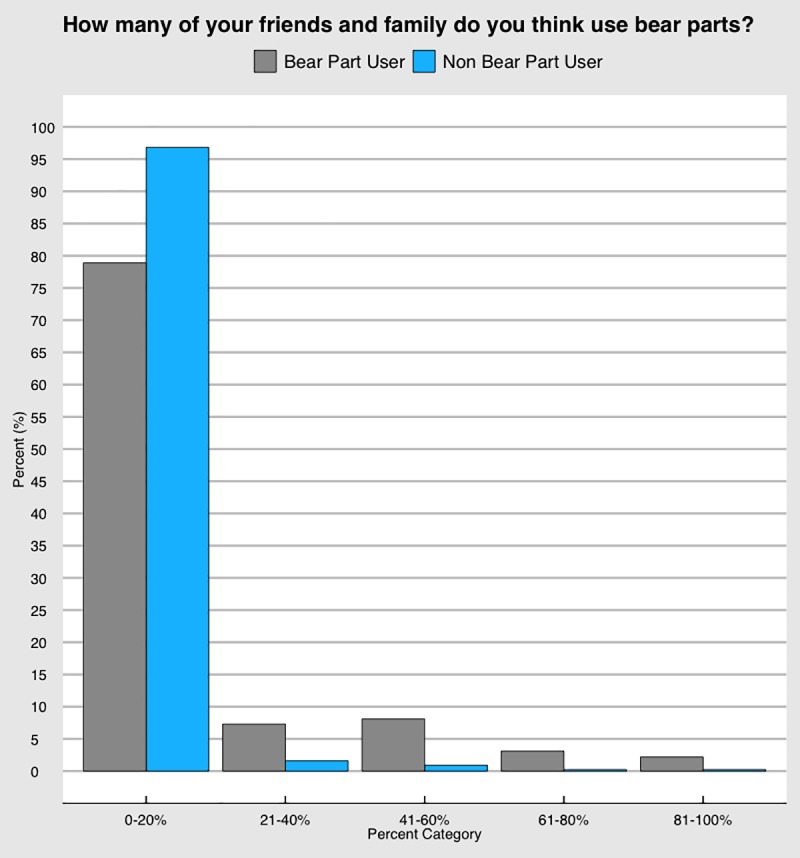
False consensus estimates for all respondents. Use of bear parts was calculated as being any individual who directly admitted to having ever used any bear part. There was significant difference between bear part users and non-bear part users in belief of their social group’s use of bear parts (n = 1,934).

Respondents were classified as either bear part users or non-bear part user according to their self-reported use of any bear part. A chi-squared test on the false consensus estimates found statistically significant differences between the observed and expected values of each group, users and non-users (df = 4, p = 0.0039). Bear part users were more likely to believe that more of their social group had used bear parts than did non-bear part users (Fig 4). However, both groups were still more likely to believe that of their social group, only 0–20% of the individuals used bear parts. This aligns with the prevalence estimates found for direct questioning, UCT, and RRT. NT was found to be slightly higher in both Stung Treng and the Cardamom Mountains than the FCB estimate, although the NT estimate from Phnom Penh was supported by the FCB result.

## Discussion

Globally, the use of animals for traditional medicine is reportedly as high as 80%, yet studies that aim to understand and contextualise such use are still lacking [48]. This is important in light of the noted decline in species worldwide, often for medical purposes [49, 50]. Moreover, understanding and changing this use can be difficult, as the use of animals in traditional medicine is often an integral part of a culture [6]. This is likely true of bear bile/gallbladder within traditional Chinese medicine, yet it is less certain how intrinsic the use of bear bile/gallbladder is in Cambodia. The aim of this study, therefore, was to begin understanding use of bear bile/gallbladder in a Cambodian context, by obtaining a general understanding of the prevalence of bile/gallbladder consumption, through the use of specialised questioning techniques (SQTs).

### Specialised questioning techniques in Cambodia

An additional objective of this study was to understand whether SQTs such as randomised response technique (RRT), unmatched count technique (UCT), nominative technique (NT), and false consensus bias (FCB) could be useful in a Southeast Asian setting, and for a conservation issue like illegal wildlife consumption. We found that individuals in our sample were being influenced by FCB. When we separated our sample into users of bear parts and non-users, users of bear parts were statistically significantly more likely to believe that their social group had a higher prevalence of bear part use, compared to the beliefs held by non-bear part users. However, it is worth noting that the majority of both users and non-users of bear parts believed that the prevalence of use was somewhere between 0–20% of their social circle. As shown in Fig 3, this belief correlates with the average prevalence estimates found both in direct questioning and for the other three SQTs.

Respondents in all three sites overwhelmingly indicated trust in RRT over UCT, when asked which of the methods they felt best protected their answer. However, as the results indicate, respondents answered deceitfully for RRT (Fig 2). This superficially paradoxical result could be a result of respondents trusting the method to shield them enough to lie, or to not-comply with the "forced" response. Respondents may have felt uncomfortable admitting to something they hadn't done when the role of the die required a yes response. It is also possible that, as the method was used last in the interviews, respondents may have wanted to maintain consistency by giving the same answer as they gave when directly asked about bear part use, whether that answer was true or not. The respondents’ desire to maintain face may also have played a role in that respondents may not have felt comfortable admitting that they did not understand the instructions for RRT when first explained [51].

In contrast to the RRT results, we found that the prevalence estimate of UCT was higher than direct questioning in all locations, despite respondents’ stated distrust of the test. However, we noted two problems with UCT as used in this study which suggest that the UCT results may not be precise. One is the sample size. Nuno et al. [30] sampled over 1000 individuals, yet still found significant statistical error in their results. As we were unable to sample over 1000 individuals in each specific location, the 15.2% bear part usage rate obtained for the entire country (n = 1,934) may be the most robust. This result also serves to smooth out some of the variability found in the responses between each sample site. In UCT the expected distribution of responses is to have the majority of responses grouped around 2–3 behaviours [31]. In this study, individuals in Stung Treng responded most similarly to the expected UCT distribution. However, in the Cardamom Mountains and Phnom Penh, respondents in both the control and treatment groups answered with much higher instances of “0” than would be expected to occur. The UCT questions did not undergo extensive pre-testing, which may have affected applicability of the responses to the sample.

Results from NT were significantly higher overall than any of the other results (Fig 2). However, the questions used for NT were less specific than other questions. All other questions asked only about bear bile and bear gallbladder, whereas NT asked about all bear parts used for all purposes. Therefore, it is reasonable that NT is much higher than the other results. Those communities that are more likely to use more other parts of the bear, such as bear meat, would naturally have higher behaviour prevalence. This is supported by findings in our study where 22.5% of the population in the Cardamom Mountains had consumed bear meat, versus 1.7% in Phnom Penh and 6.9% in Stung Treng. At the same time, the high prevalence in Stung Treng could be explained by the disparate time frame, as our NT question also asked about bear part use over life-time, rather than over last 12 months, as the other questions did.

It is clear that although deceit was a factor when responding to the SQTs, individuals still admitted to use of bear bile and gallbladder at a significantly higher rate than they did when asked directly about their use. Furthermore, our results indicate that in Cambodia bear bile/gallbladder usage is not confined to rural areas, but is also present in urban centres, although the reliability of RRT in particular was very low in the urban context. This may indicate greater social desirability bias, a hypothesis supported by the direct estimate for Phnom Penh. Phnom Penh usage as measured by SQT was found to be just as prevalent as bear part usage in rural locations, but direct admittance of that use was significantly lower than in rural locations. This result could also have been influenced by the dynamics of illegal wildlife consumption within Phnom Penh. If wildlife consumption in the urban centre of Cambodia is correlated to social status, then the lower prevalence estimates may reflect under-sampling of the elite, due to the constraints of the sampling strategy. Additionally, the majority of bear part users from Phnom Penh who directly admitted to use were likely to have consumed and/or obtained bear parts in Phnom Penh City. However, some cited rural provinces as the places either they obtained or consumed bear parts.

Although there was significant variability between all three techniques, our results indicate that RRT is a non-effective method in Cambodia. It is expected that SQTs obtain a prevalence estimate higher or equal to the direct questions [25] (although for some behaviours it is possible that the reverse is true [52]). In this study RRT was significantly lower than all other methods in 2/3 sites. Although individuals stated that they trusted the method, the 0% prevalence estimate found in the Cardamom Mountains, and the generally low estimates found at both other sites, indicates that it is not effective in the Cambodian setting. As discussed previously, this inefficacy could be for many reasons, including lack of understanding due to cultural factors, or such great trust in RRT over UCT that individuals felt comfortable lying, with the understanding that the interviewer would not know that they had lied.

By comparison, we found generally trustworthy results for UCT in two of our study sites of Stung Treng and Phnom Penh. However, the high amount of “0” responses in the Cardamom Mountains for UCT may indicate significant distrust with the method. One possible explanation is that UCT may be ineffective in rural areas in Cambodia, particularly considering the problems Ibbett et al. [31] found in their study. Although UCT performed well in Stung Treng, the least densely populated province in Cambodia, the interviews themselves were performed around the provincial town centre, an area of relatively moderate urbanity. Additionally, we suspect that UCT may be ineffective in areas that have been exposed to frequent surveys. As a biodiversity hotspot, the Cardamom Mountains are a popular site for research in Cambodia, and subject to greater wildlife law enforcement and habitat protection [53]. This may have an effect by causing individuals in those areas to be wary about researchers, whose research has potentially led, or are perceived to have led, to greater restrictions on resources. Another explanation for UCT’s apparent inefficacy in the Cardamom Mountains is the findings of Tschuyia et al. [54], who argued that error within UCT occurs when a behaviour is relatively rare in a population. However, this seems to be an inadequate explanation for the effect seen, as UCT appeared to work well in Phnom Penh and Stung Treng, where the behaviour was at a similar prevalence, according to the other methods used. The technique that appeared to be most reliable across sites was NT. Because it was not specifically highlighted as being separate from the other questions in the survey, we suspect most individuals were comfortable in answering those questions.

Future studies that aim to test these techniques should be created to ask exactly the same question with the same time frame, so as to be fully comparable. However, we are able to draw some important conclusions. First, RRT does not appear to be effective for asking research questions in a Cambodian context, and we would not recommend its continued use in the region. Second, despite the variability in question format, use of bear parts appears to be much higher than was previously thought, based on the UCT and NT results, when taken together. Additionally, individuals are evidently shielding their behaviour when asked. As we found knowledge regarding the illegality of bear part use to be fairly high in the population, this deceit in answering direct questions could be due primarily to worries over illegality, rather than concerns over social undesirability.

Finally, we noticed that FCB seemed to work well in the population, but future studies would benefit from greater differentiation in the answer categories. As predicted, non bear-part users estimated that a lower proportion of their family/friends use bear parts. However, from our data we do not know the precise proportion of family/friends that respondents estimated use bear parts. Based on our results we now know that a finer scale is required, particularly between 0 and 20%.

In particular, we propose two key readjustments of the category of 0–20%. First, the category of “0%” should be separated into its own category, so as to reflect those individuals who exist (or believe they exist) within a social group that does not use bear parts at all. Secondly, the perceived percentages of use (i.e. 1% and up) should be divided into smaller categories. Both of these adjustments will assist in aligning with the “true” prevalence of use in Cambodia, which could be as high as 20% in some locations.

### Conservation implications of illegal bear part consumption in Cambodia

There is great potential for specialised questioning techniques to provide more accurate understanding of sensitive behaviours and therefore to inform robust policy decisions and evaluate conservation interventions such as demand reduction and behaviour change efforts [25, 28]. As a result, studies such as this are essential for informing subsequent conservation research in myriad geographical contexts.

Cambodia's protected landscapes harbour regionally significant populations of bears [37]. If the “true” prevalence estimate is assumed to be somewhere between the average of the SQTs for all three sites and the average for direct questions between all three sites, then there is considerable demand for bear parts within Cambodia, which represents a serious threat to the ongoing survival of potentially regionally important bear populations.

The high level of demand from within Cambodia presents challenges for effective wildlife conservation. In Cambodia, and throughout the region, illegal hunting of terrestrial wildlife is often carried out using snares which can be difficult to detect during law enforcement patrols and which can be manufactured and set easily without significant financial or opportunity costs on the part of the hunter [55, 56]. Low investment costs and low risk of punishment, coupled with the potentially high profit margins from the capture and sale of high-value species such as bears, mean that there is little disincentive to poaching. In addition to improved wildlife law enforcement, continued survival of Cambodia's remaining wildlife will require effective demand reduction and behaviour change interventions.

## Supporting information

S1 Data Cambodian dataset. All relevant data collected for this study.
(XLSX)

S1 Questionnaire Cambodia questionnaire. The questionnaire used to gather the data.
(DOCX)

## References

[1] Mendenhall CD, Daily GC, Ehrlich PR. Improving estimates of biodiversity loss. Biological Conservation. 2012 7 1;151(1):32–4.

[2] Sodhi N.S., Koh L.P., Brook B.W. and Ng P.K., 2004 Southeast Asian biodiversity: an impending disaster. Trends in Ecology & Evolution, 19(12): 654–660 16701328 10.1016/j.tree.2004.09.006

[3] Duffy R, St John FA, Büscher B, Brockington DA. The militarization of anti-poaching: undermining long term goals?. Environmental Conservation. 2015 12;42(4):345–8.

[4] Alves RR, Rosa IL, Albuquerque UP, Cunningham AB. Medicine from the wild: an overview of the use and trade of animal products in traditional medicines In: Animals in traditional folk medicine 2013 (pp. 25–42). Springer, Berlin, Heidelberg.

[5] Ogada D, Shaw P, Beyers RL, Buij R, Murn C, Thiollay JM, Beale CM, Holdo RM, Pomeroy D, Baker N, Krüger SC. Another continental vulture crisis: Africa's vultures collapsing toward extinction. Conservation Letters. 2016 3;9(2):89–97.

[6] Alves RR, Silva JS, da Silva Chaves L, Albuquerque UP. Ethnozoology and animal conservation. Ethnozoology 2018 (pp. 481–496).

[7] Van Vliet N, Moreno J, Gomez J, Zhou W, Fa JE, Golden C, Nobrega Alves RR, Nasi R. Bushmeat and human health: Assessing the Evidence in tropical and sub-tropical forests. Ethnobiology and Conservation. 2017 4 20;6(3):1–45.

[8] Lee TM, Sigouin A, Pinedo-Vasquez M, Nasi R. The harvest of wildlife for bushmeat and traditional medicine in East, South and Southeast Asia: Current knowledge base, challenges, opportunities and areas for future research. CIFOR; 2014 11 11.

[9] Burke A, Wong YY, Clayson Z. Traditional medicine in China today: implications for indigenous health systems in a modern world. American Journal of Public Health. 2003 7;93(7):1082–4. 12835188 10.2105/ajph.93.7.1082 PMC1447912

[10] Feng Y, Siu K, Wang N, Ng KM, Tsao SW, Nagamatsu T, et al Bear bile: dilemma of traditional medicinal use and animal protection. Journal of ethnobiology and ethnomedicine. 2009 12;5(1):2. 19138420 10.1186/1746-4269-5-2 PMC2630947

[11] Mainka SA, Mills JA. Wildlife and traditional Chinese medicine: supply and demand for wildlife species. Journal of zoo and wildlife medicine. 1995 6 1:193–200.

[12] Knechtges DR. A literary feast: Food in early Chinese literature. Journal of the American Oriental Society. 1986 1 1;106(1):49–63.

[13] Kemmerer L. Bear Basics. Bear Necessities: Rescue, Rehabilitation, Sanctuary, and Advocacy. 2015 Aug 27:17.

[14] Sethy J, Chauhan NP. Use and trade of bear body parts: Impact and conservation in Arunachal Pradesh state, India. International Journal of Bio-resource and Stress Management. 2011;2(4):409–15.

[15] Dutton AJ, Hepburn C, Macdonald DW. A stated preference investigation into the Chinese demand for farmed vs. wild bear bile. PloS one. 2011 7 20;6(7):e21243 10.1371/journal.pone.0021243 21799733 PMC3140486

[16] Liu F, McShea WJ, Garshelis DL, Zhu X, Wang D, Shao L. Human-wildlife conflicts influence attitudes but not necessarily behaviors: Factors driving the poaching of bears in China. Biological Conservation. 2011 1 1;144(1):538–47.

[17] Scotson, L, Fredriksson, G, Augeri, D, Cheah, C, Ngoprasert, D, Wai-Ming, W. Helarctos malayanus. The IUCN Red List of Threatened Species. 2017: e.T9760A45033547. Available from 10.2305/IUCN.UK.2017-3.RLTS.T9760A45033547.en. Downloaded on 05 April 2018.

[18] Garshelis, D, Steinmetz, R. Ursus thibetanus. (errata version published in 2017) The IUCN Red List of Threatened Species. 2016: e.T22824A114252336. 10.2305/IUCN.UK.2016-3.RLTS.T22824A45034242.en. Downloaded on 01 December 2017

[19] Burgess EA, Stoner S, Foley KE. Brought to Bear: An Analysis of Seizures Across Asia (2000–2011): a TRAFFIC Report. TRAFFIC; 2014.

[20] Livingstone E, Gomez L, Bouhuys J. A review of bear farming and bear trade in Lao People's Democratic Republic. Global Ecology and Conservation. 2018 3 15:e00380.

[21] Crudge B., Nguyen T., and Cao T.T. The challenges and conservation implications of bear bile farming in Vietnam. Oryx. 2018 7; 1–8.

[22] Shepherd CR, Nijman V. The trade in bear parts from Myanmar: an illustration of the ineffectiveness of enforcement of international wildlife trade regulations. Biodiversity and Conservation. 2008 1 1;17(1):35–42.

[23] Davis EO, O'Connor D, Crudge B, Carignan A, Glikman JA, Browne-Nuñez C, et al Understanding public perceptions and motivations around bear part use: A study in northern Laos of attitudes of Chinese tourists and Lao PDR nationals. Biological conservation. 2016 11 1;203:282–9.

[24] Crudge B, O'Connor D, Hunt M, Davis EO, Browne‐Nuñez C. Groundwork for effective conservation education: an example of in situ and ex situ collaboration in South East Asia. International Zoo Yearbook. 2016 1 1;50(1):34–48.

[25] St John FA, Edwards-Jones G, Gibbons JM, Jones JP. Testing novel methods for assessing rule breaking in conservation. Biological Conservation. 2010 4 1;143(4):1025–30.

[26] Nuno A, St John FA. How to ask sensitive questions in conservation: A review of specialized questioning techniques. Biological Conservation. 2015 9 1;189:5–15.

[27] Dawes RM. Statistical criteria for establishing a truly false consensus effect. Journal of Experimental Social Psychology. 1989 1 1;25(1):1–7.

[28] St John FA, Keane AM, Edwards-Jones G, Jones L, Yarnell RW, Jones JP. Identifying indicators of illegal behaviour: carnivore killing in human-managed landscapes. Proceedings of the Royal Society of London B: Biological Sciences. 2011 7 27:rspb20111228. 10.1098/rspb.2011.1228 PMC3248731 21795272

[29] St John FA, Mai CH, Pei KJ. Evaluating deterrents of illegal behaviour in conservation: carnivore killing in rural Taiwan. Biological Conservation. 2015 9 1;189:86–94.

[30] Nuno AN, Bunnefeld N, Naiman LC, Milner‐Gulland EJ. A novel approach to assessing the prevalence and drivers of illegal bushmeat hunting in the Serengeti. Conservation Biology. 2013 12 1;27(6):1355–65. 10.1111/cobi.12124 24001112 PMC4232883

[31] Ibbett H, Lay C, Phlai P, Song D, Hong C, Mahood SP, et al Conserving a globally threatened species in a semi-natural, agrarian landscape. Oryx. 2017 5:1–1.

[32] Hinsley A, Nuno A, Ridout M, St John FA, Roberts DL. Estimating the Extent of CITES Noncompliance among Traders and End‐Consumers; Lessons from the Global Orchid Trade. Conservation Letters. 2017 9 1;10(5):602–9.

[33] Johnson T., & van de Vijver F. J. Social desirability in crosscultural research In Harness J., van de Vijver F. J., & Mohler P. (Eds.), Cross-cultural survey methods. 2002 (pp. 193–202). New York: Wiley

[34] McPherson M, Smith-Lovin L, Cook JM. Birds of a feather: Homophily in social networks. Annual review of sociology. 2001 8;27(1):415–44.

[35] Miller JD. The nominative technique: A new method of estimating heroin prevalence. NIDA Research Monograph. 1985;54:104–24. 3929108

[36] Newing H. Conducting research in conservation: social science methods and practice Routledge; 2010 10 18.

[37] The World Factbook. 2018. Washington, DC: Central Intelligence Agency, 2017. Accessed 22 June 2018 https://www.cia.gov/library/publications/the-world-factbook/index.html

[38] Gray TN, Billingsley A, Crudge B, Frechette JL, Grosu R, Herranz-Muñoz V, et al Status and conservation significance of ground-dwelling mammals in the Cardamom Rainforest Landscape, southwestern Cambodia. Cambodian Journal of Natural History. 2017;2017:38–48.

[39] Coudrat CN, Rogers LD, Nekaris KA. Abundance of primates reveals Samkos Wildlife Sanctuary, Cardamom Mountains, Cambodia as a priority area for conservation. Oryx. 2011 7;45(3):427–34.

[40] Singh S, Boonratana R, Bezuijen M, Ko S. Trade in natural resources in Stung Treng Province, Cambodia: An assessment of the wildlife trade. TRAFFIC, MWBP, Vientiane, Lao PDR. 2006.

[41] Shatkin G. ‘Fourth World’cities in the global economy: The case of Phnom Penh, Cambodia. International Journal of Urban and Regional Research. 1998 9 1;22(3):378–93.

[42] Lensvelt-Mulders GJ, Hox JJ, Van Der Heijden PG. How to improve the efficiency of randomised response designs. Quality and Quantity. 2005 6 1;39(3):253–65.

[43] Coutts E, Jann B. Sensitive questions in online surveys: Experimental results for the randomized response technique (RRT) and the unmatched count technique (UCT). Sociological Methods & Research. 2011 2;40(1):169–93.

[44] Team R Core. R: A language and environment for statistical computing. R Foundation for Statistical Computing, Vienna, Austria 2016.

[45] Wickham H. ggplot2: Elegant Graphics for Data Analysis. Springer-Verlag New York 2009.

[46] National Institute of Statistics, Ministry of Planning. "General Population Census of Cambodia 2008—Provisional population totals" (PDF). 2008.

[47] Galobardes B. and Demarest S. Asking sensitive information: an example with income. Sozial-und Präventivmedizin. 2003 48(1): 70–72. 12756891 10.1007/s000380300008

[48] Alves RR, das Graças Gerônimo Oliveira M, Barboza RR, Lopez LC. An ethnozoological survey of medicinal animals commercialized in the markets of Campina Grande, NE Brazil. Human Ecology Review. 2010 7 1:11–7.

[49] Corlett RT. The impact of hunting on the mammalian fauna of tropical Asian forests. Biotropica. 2007 5;39(3):292–303.

[50] Alves RR, Rosa IM. Biodiversity, traditional medicine and public health: where do they meet?. Journal of ethnobiology and ethnomedicine. 2007 12;3(1):14. 17376227 10.1186/1746-4269-3-14 PMC1847427

[51] Gellman M. World views in peace building: a post-conflict reconstruction challenge in Cambodia. Development in practice. 2010 2 1;20(1):85–98.

[52] Barnett J. Sensitive questions and response effects: an evaluation. Journal of Managerial Psychology. 1998 2 1;13(1/2):63–76.

[53] Nicholas J, Simpson V, Mould A, Eames JC, Gray TN, Sinclair R, et al Will the recent changes in protected area management and the creation of five new protected areas improve biodiversity conservation in Cambodia?. Cambodian Journal of Natural History. 2016:1.

[54] Tsuchiya T, Hirai Y, Ono S. A study of the properties of the item count technique. Public Opinion Quarterly. 2007 1 1;71(2):253–72.

[55] Harrison RD, Sreekar R, Brodie JF, Brook S, Luskin M, O'Kelly H, et al Impacts of hunting on tropical forests in Southeast Asia. Conservation Biology. 2016 10 1;30(5):972–81. 10.1111/cobi.12785 27341537

[56] Gray TN, Hughes AC, Laurance WF, Long B, Lynam AJ, O’Kelly H, et al The wildlife snaring crisis: an insidious and pervasive threat to biodiversity in Southeast Asia. Biodiversity and Conservation. 2018 3 1;27(4):1031–7.

