# Correction: Understanding the prevalence of bear part consumption in Cambodia: A comparison of specialised questioning techniques

**DOI:** 10.1371/journal.pone.0214392

**Published:** 2019-03-20

**Authors:** Elizabeth Oneita Davis, Brian Crudge, Thona Lim, David O’Connor, Vichet Roth, Matt Hunt, Jenny Anne Glikman

There are errors in the author affiliations. The correct affiliations are as follows:

Elizabeth Oneita Davis^1^, Brian Crudge^2^, Thona Lim^2^, David O’Connor^1,3,4^, Vichet Roth^2^, Matt Hunt^2^, Jenny Anne Glikman^1^

**1** San Diego Zoo Institute for Conservation Research, Escondido, CA, USA, **2** Free the Bears, Phnom Penh, Cambodia, **3** National Geographic Partners, NW, Washington DC, USA, **4** The Faculty of Biological Sciences, Goethe University, Frankfurt am Main, Germany.
