# Correction: Understanding the prevalence of bear part consumption in Cambodia: A comparison of specialised questioning techniques

**DOI:** 10.1371/journal.pone.0233798

**Published:** 2020-05-20

**Authors:** Elizabeth Oneita Davis, Brian Crudge, Thona Lim, David O’Connor, Vichet Roth, Matt Hunt, Jenny Anne Glikman

In the ‘Specialised questioning techniques in Cambodia’ subsection of the Discussion, there is an error in the first sentence of the fourth paragraph. The correct sentence is: The standard error for NT overlapped with UCT in Phnom Penh (Fig 2). In both other sites the estimated prevalence estimates for NT were significantly higher than the other techniques.

**Fig 2:**
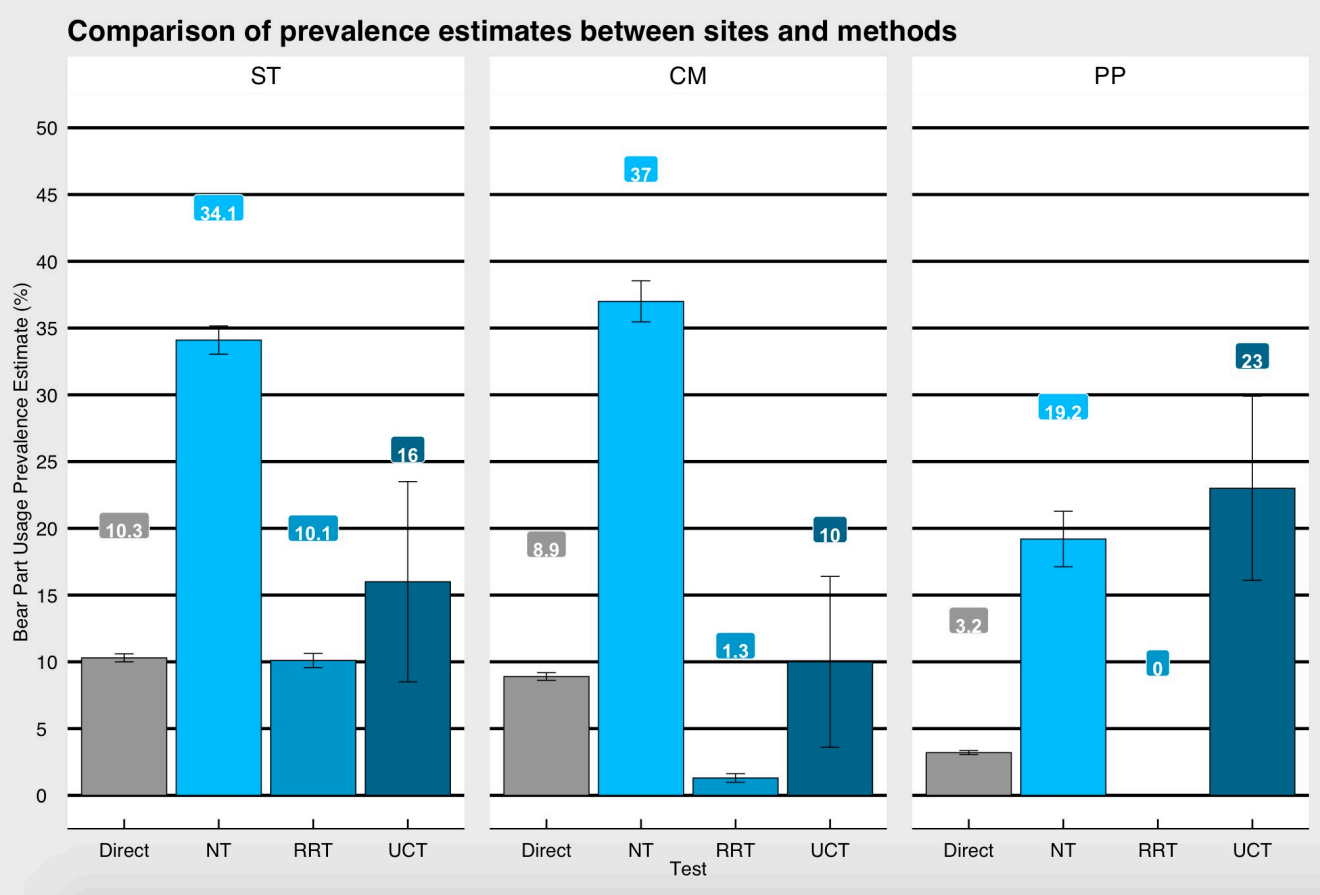
Prevalence estimates. Prevalence estimates of bear part use obtained through specialised questioning techniques (SQTs) and direct questioning for all three field sites (Stung Treng (ST): n = 641; Cardamom Mountains (CM): n = 638; Phnom Penh (PP): n = 649.

There is also an error in the first sentence of the seventh paragraph of this subsection. The correct sentence is: By comparison, we found generally trustworthy results for UCT in two of our study sites of Stung Treng and Phnom Penh, barring the larger standard errors and by extension variability that characterizes UCT (e.g. [43]).

The second sentence of the eighth paragraph of this subsection does not appear. The second sentence is: Additionally, UCT’s high variability suggests that alternative techniques such as NT may be more useful in gaining precise estimates.

In Fig 2, the unmatched count technique (UCT) and standard error calculations are incorrect. Please see the correct Fig 2 here.
