# Supplementary material for: Understanding the prevalence of bear part consumption in Cambodia: A comparison of specialised questioning techniques
Source: PLoS One. 2019 Feb 20;14(2):e0211544. doi: 10.1371/journal.pone.0211544 (PMC6382108; doi:10.1371/journal.pone.0211544)
Supplement: S1 Questionnaire — The questionnaire used to gather the data. (DOCX) [file pone.0211544.s002.docx]

***!!! Make sure the participant lives in Phnom Penh and has lived in Phnom Penh for at least 2 years !!!***

**Wildlife Survey**

*We would like to ask you for the following demographic information to help us make general conclusions. Your responses will remain completely secret, confidential and will be anonymised.*

***Please do not write respondent’s name, address or contact details anywhere on the form***

1. Gender

**Male**

**Female**

1. What is your age? **____________ years**
2. Month of birth? __________ or **don't know**
3. **a.** What is the **highest** level of education you have completed? *Please tick one box*

**None**

**Literacy certificate**

**Part of Primary school**

**Primary school**

**Secondary school**

**High school**

**Bachelors**

**Graduate Degree (Masters, Law, PhD)**

**b.** Where did you receive your highest level of education? *Please tick one box*

**Cambodia**

**Elsewhere in Asia**

**Outside Asia**

1. What is your nationality? (tick one box)

**Cambodian**

**Other: ____________________________**

1. What is your ethnicity? *(You may tick more than one box. Please tick all boxes that apply.)*

**Khmer**

**Cham**

**Chinese**

**Thai**

**Lao (Lao Loum)**

**Lao (other)** *Please state***________________________________**

**Vietnamese (Kinh)**

**Vietnamese (other)** *Please state***_________________________**

**Other:** *Please state***___________________________________**

1. What is your religion? (*You may tick more than one box. Please tick all boxes that apply.)*

**None**

**Ancestor spirits**

**Buddhist**

**Christian**

**Confucian**

**Jewish**

**Muslim**

**Taoist**

**Other: *Please state*_________________________**

1. **a.** Where did you grow up?

*(Where did you spend most of your childhood? This may be different from the place where you were if you moved and lived most of your childhood in a different place.)*

**Province/Municipality:_____________________________ District/District:__________________**

**8.b**. Where do you live now?

**Province/Municipality:_______________________ District/District:_________________________**

**8.c.** How long have you lived there? **_____________years**

***(****Round to the nearest year. For example, for someone who lived in a place for 1 year and 1-6 months, write "1". For someone who lived in a place for 1 year 7-11 months, write "2".)*

1. Whose opinions do you value most in your life for advice on important matters?

Please rank the different groups from 1-5 (*1 is highest ranked, 2 is second highest ranked and 3 is third highest ranked, etc.*)

**___­­­­­­­­­ ­­ Family (e.g. mother, father, sisters, brothers, husband/wife, grandparents, uncles, aunts, cousins)**

**___­­­­­­­­­ ­­ Friends (e.g. boyfriend, girlfriend, close friend)**

**___­­­­­­­­­ ­­ Workmates or bosses**

**___­­­­­­­­­ ­­ Medical experts (e.g. traditional healers, doctors, nurses)**

**___­­­­­­­­­ ­­ Monks and Religious leaders**

1. Please indicate how much you value the work of the below listed people. *<Please read the options, "Don't value at all" to "High Value" or "Don't know"> Please circle one box in each row*

*Use printed table and a stone. Ask respondent to place stone in their choice for each question on the printed table. Remove stone between questions.*

| **a. Traditional healer** (Khmer, Chinese, Lao, etc.) | Don’t value at all | Value a little | Value moderately | Highly value | Don’t know |
| --- | --- | --- | --- | --- | --- |
| **b. Western medical expert** (e.g. doctor, nurse, pharmacist) | Don’t value at all | Value a little | Value moderately | Highly value | Don’t know |
| **c. Religious leader/Monk** (e.g. Christian leader, Muslim leader, Monk) | Don’t value at all | Value a little | Value moderately | Highly value | Don’t know |
| **d. Cambodian Forestry Service** (e.g. government or protected area staff) | Don’t value at all | Value a little | Value moderately | Highly value | Don’t know |
| **e. Conservation worker** | Don’t value at all | Value a little | Value moderately | Highly value | Don’t know |
| **f. Traffic police** | Don’t value at all | Value a little | Value moderately | Highly value | Don’t know |

# Which of the following do most medical practitioners you know of use: (*Tick one box)*

**Western medicine**

**Traditional medicine**

**A combination of western and traditional medicine**

**Don’t know**

1. **a.**  In the past 12 months did you use Western medicine? (*Tick one box*)

**Yes No**

b. In the past 12 months, did you use Traditional medicine? (*Tick one box*)

**Yes No**

c. In the past 12 months, which did you use more? (*Tick one box*)

**Western medicine**

**Traditional medicine**

**Other:_______________**

**Don’t know**

**UCT Section**

*UTC: Green (A) / Red (B)*

***<I am going to use a game with cards to ask about activities that people do. The method ensures that your answers are completely anonymous. Each time I will show you a card, and you look at the list of things on it. I will then ask HOW MANY of these things you have done over the past 12 months. I don't want to know which ones, just how many.>***

1. *I will start with a question on animals to show you how the method works. Can you to tell me* ***how many*** *of these animals you have seen in real life (not on TV, facebook, computer or phone) over the past 12 months? Please do not tell me which ones you have seen.*

*Answer:*

1. The next list is about transportation. In the past year, how many on this list have you done? *Do not state which you have or have not done. Just how many.*

*Answer:*

1. The next list is about medical treatments. In the past year, how many of the following have you done? *Do not state which you have or have not done. Just how many.*

*Answer:*

**<OK that is the end of the card game>**

1. **a.** Thinking of your closest family and friends, what percentage (between 0-100%) of them do you think use bear parts or products for medicine or other purposes?

*Please circle one below – if you don’t know, please guess.*

**0-20% 21-40% 41-60% 61-80% 81-100%**

**16 b.** How many of your close friends do you know for certain have used/consumed bear parts for medicine or other purposes?

**Answer:____________** *(if “0” write N/A and skip to 17. If “1”, go to question 16 c. If more than one, ask the interviewee to write down their friends' initials on pieces of paper and then select one friend. Note: the pieces of paper will be disposed of. Then ask question 16 c in relation to that one person).*

**16 c.** Other than you, how many other people do you believe know that the nominated friend has used bear parts or products for medicine or other purposes?

**Answer:____________**

1. For *each* of the following statements, please indicate whether you think they are true or false. *(Please circle your response for each statement.)*

| 1. **The number of bears in the forest in Cambodia is increasing** | True | False | Don’t Know |
| --- | --- | --- | --- |
| 1. **Hunting bears in Cambodia is legal** | True | False | Don’t Know |
| 1. **It is possible to extract bile from a bear without killing the animal** | True | False | Don’t Know |
| 1. **Most bears in farms were born in captivity** | True | False | Don’t Know |
| 1. **Consuming bear products in Cambodia is legal** | True | False | Don’t Know |
| 1. **Most people whose opinion you value...**have used bear bile for medicine and other purposes in the past | True | False | Don’t Know |
| 1. **Most people whose opinion you value...**will use bear bile in the future | True | False | Don’t Know |
| 1. **Most people whose opinion you value...**believe you should use bear bile | True | False | Don’t Know |

1. We are interested in learning your views on bear bile.

*In this section* ***'wild bears'*** *are bears that live in the wild;* ***'farmed bears'*** *are bears that are kept in cages on a “bear bile farm” for bile extraction. Please indicate if you agree or disagree with the following statements. (Circle one response per row. Use printed table and a stone. Ask respondent to place stone in their choice for each question on the printed table. Remove stone between questions.)*

| 1. **Bear bile has medicinal value** | Strongly Disagree | Disagree | Neither agree nor disagree | Agree | Strongly Agree | Don’t know |
| --- | --- | --- | --- | --- | --- | --- |
| 1. **Bile from wild bears has stronger medicinal properties than bile from farmed bears** | Strongly Disagree | Disagree | Neither agree nor disagree | Agree | Strongly Agree | Don’t know |
| 1. **It is easy to find places to buy bear bile** | Strongly Disagree | Disagree | Neither agree nor disagree | Agree | Strongly Agree | Don’t know |
| 1. **There are good medicinal alternatives to bear bile/bear gallbladder** | Strongly Disagree | Disagree | Neither agree nor disagree | Agree | Strongly Agree | Don’t know |
| 1. **The use of bear bile is an important part of your culture** | Strongly Disagree | Disagree | Neither agree nor disagree | Agree | Strongly Agree | Don’t know |
| 1. **Using bile from wild bears will lead to the extinction of bears in the wild** | Strongly Disagree | Disagree | Neither agree nor disagree | Agree | Strongly Agree | Don’t know |
| 1. **It is acceptable to use bile from bears that are farmed** | Strongly Disagree | Disagree | Neither agree nor disagree | Agree | Strongly Agree | Don’t know |

1. Have you ever consumed/used any of the following bear parts or products? *(Please circle one answer on each row)*

| 1. Bear paw soup | Yes | No | Don’t know |
| --- | --- | --- | --- |
| 1. Bear paw rice wine | Yes | No | Don’t know |
| 1. Bear bile | Yes | No | Don’t know |
| 1. Bear gallbladder | Yes | No | Don’t know |
| 1. Bear fat | Yes | No | Don’t know |
| 1. Bear blood | Yes | No | Don’t know |
| 1. Bear bone | Yes | No | Don’t know |
| 1. Bear meat | Yes | No | Don’t know |

1. When was the most recent time you used bear parts or products? *Please tick one box*

**Never**

**Within the last year**

**Between 1-5 years ago**

**Between 6-10 years ago**

**More than 10 years ago**

**Don’t know**

1. How often have you consumed bear parts or products in your lifetime? *Please circle one below*

**Never**

**1-10 times**

**11-20 times**

**21-30 times**

**more than 30 times**

**Don’t know**

1. **a.** If you have consumed bear parts, who offered/recommended it to you the first time?

**n/a**

**relative**

**friend**

**acquaintance**

**medical expert**

**other:____________________**

**b.** Where were you?

**village**

**city**

**other:____________________**

**c. What was your main reason for being in location (when you consumed bear parts)?**

**____________________________________________________________________________**

1. If you had to buy and cost was not an issue, which product would you prefer to buy of the following pairs? *(Please tick* ***one*** *box* ***in each pair. Synthetic bear bile is made from chemicals in a laboratory to act like the medicinal component in natural bear bile.****)*

| Bear gallbladder Bear paw |
| --- |
| Bear bile Bear gallbladder |
| Wild bear bile Farmed bear bile |
| Farmed bear bile Synthetic bear bile |
| Wild bear bile Synthetic bear bile |
| Wild bear bile Herbal medicine |
| Farmed bear bile Herbal medicine |
| Herbal medicine Western medicine |
| Wild bear bile Western medicine |
| Farmed bear bile Western medicine |

| 1. **Were you or someone in your family ill with any of the following ailments in the past 12 months? (Please circle all that apply)** | | | 1. **For each ailment that, what treatments did you use?** *(Please circle all treatments used for each ailment experienced in past 12 months. Then ask respondent if they used any of the other approaches.)* | | | | | |
| --- | --- | --- | --- | --- | --- | --- | --- | --- |
| **Sores** | Yes | No | herbal medicine | western medicine | synthetic bear bile | farmed bear bile | wild bear bile | Other: |
| **Hemorrhoids** | Yes | No | herbal medicine | western medicine | synthetic bear bile | farmed bear bile | wild bear bile | Other: |
| **Sore throat** | Yes | No | herbal medicine | western medicine | synthetic bear bile | farmed bear bile | wild bear bile | Other: |
| **Sprains** | Yes | No | herbal medicine | western medicine | synthetic bear bile | farmed bear bile | wild bear bile | Other: |
| **Bruising** | Yes | No | herbal medicine | western medicine | synthetic bear bile | farmed bear bile | wild bear bile | Other: |
| **Muscle ailments** | Yes | No | herbal medicine | western medicine | synthetic bear bile | farmed bear bile | wild bear bile | Other: |
| **Epilepsy** | Yes | No | herbal medicine | western medicine | synthetic bear bile | farmed bear bile | wild bear bile | Other: |
| **Liver disease** | Yes | No | herbal medicine | western medicine | synthetic bear bile | farmed bear bile | wild bear bile | Other: |
| **Internal bruising or hematoma** | Yes | No | herbal medicine | western medicine | synthetic bear bile | farmed bear bile | wild bear bile | Other: |

24.10 Were you or someone in your family pregnant or have a baby in the last 12 months?

Yes No

- 1. Which medicines did you/she use during pregnancy or after childbirth? Please tick all that apply.

herbal medicine western medicine synthetic bear bile

farmed bear bile wild bear bile Other:__________________

**RRT** <explain the rules of the RRT game, and try the practice questions>

***SHAKE THE DICE***

1. Have you **ever** used bear bile or gallbladder?

I have [1]

I have not [2]

***SHAKE THE DICE***

2. Have you **ever** eaten or drank bear paw soup or wine?

I have [1]

I have not [2]

***SHAKE THE DICE***

3. Have you used bear bile or gallbladder **in the last 12 months**?

I have [1]

I have not [2]

***SHAKE THE DICE***

4. Have you eaten or drank bear paw soup or wine **in the last 12 months**?

I have [1]

I have not [2]

1. Do you know what a saiga (animal) is? *(Circle one*)

Yes No

1. Please choose and rank (mark 1, 2 and 3) the three wildlife products that people prefer to use to show high status and class: *(1 is highest ranked, 2 is second highest ranked, 3 is third highest ranked)*

______ Rhino horn

______ Ivory

______ Tiger skin

______ Bear bile

______ Pangolin scales

______ Saiga horn

______ Tortoise shell

**THIS IS THE END OF THE QUESTIONNAIRE. ASK THE INTERVIEWEE FOR FEEDBACK.**

1. Which section did you find easier to understand?

**UCT (the game with the cards)**  or **RRT (the game with the dice)**

1. Which section did you trust protected your answers better?

**UCT (the game with the cards)**  or **RRT (the game with the dice)**

**THANK YOU FOR YOUR PARTICIPATION.**
